# Hybrid Total Knee Arthroplasty Revisited: Midterm Followup of Hybrid versus Cemented Fixation in Total Knee Arthroplasty

**DOI:** 10.1155/2013/854871

**Published:** 2013-09-25

**Authors:** Christopher E. Pelt, Jeremy M. Gililland, Justin Doble, Benjamin M. Stronach, Christopher L. Peters

**Affiliations:** ^1^Department of Orthopaedic Surgery, University of Utah, 590 Wakara Way, Salt Lake City, UT 84108, USA; ^2^Department of Orthopaedics, University of Mississippi, 2500 North State Street, Jackson, MS 39216, USA

## Abstract

The optimal method of fixation in total knee arthroplasty is still debated. Hybrid total knee arthroplasty (TKA), with cemented tibial and cementless femoral components, is a proposed method of fixation to improve outcomes. Although several studies have shown favorable outcomes, there is still lack of consensus in the literature. We hypothesized that hybrid TKA yields similar clinical, radiographic, and survivorship results compared to fully cemented TKA. The clinical and radiographic outcomes of 304 cruciate retaining TKAs with minimum two-year followup, including 193 hybrid (mean followup of 4.1 years) and fully cemented TKAs (mean followup of 3.2 years) were evaluated. Knee society scores were similar between the two groups. The total number of femoral radiolucencies was also similar between the two groups, while a greater number of femoral Zone 4 radiolucencies were seen in the cemented group (9% versus 1.6%, *P* = 0.005). The hybrid group demonstrated a 99.2% survival rate of the femoral component out to seven years for aseptic loosening. No significant difference in survivorship was seen between the groups for all cause or aseptic failure at seven years. We conclude that hybrid fixation leads to similar intermediate-term outcomes as fully cemented components and remains a viable option in total knee arthroplasty.

## 1. Introduction

The ideal fixation in total knee arthroplasty continues to be debated. Concerns related to potential late loosening and third-body wear from cement, along with the promise of durable biologic fixation and bone preservation at revision with cementless implants, have led to the use of this alternative fixation [1–3]. Failures of the tibial component and patellar component have been shown to be problematic with cementless fixation [2, 4–6]. Many studies of hybrid fixation, with cemented tibial and patellar components and cementless femoral fixation, have shown positive results [1, 7–15]. One group reported an unacceptably high revision rate at 15 year followup and concluded that hybrid fixation should be abandoned [16, 17]. Because of the lack of consensus on this topic, we reviewed our results with hybrid knee fixation and compared them to a cohort of fully cemented knees. We hypothesized that hybrid total knee arthroplasty yields similar clinical, radiographic, and survivorship results as compared to fully cemented total knees performed over the same time period.

## 2. Methods and Materials

The study and control patients were identified from an IRB-approved prospective database. The clinical and radiographic outcomes of 261 cruciate retaining (CR) TKAs with minimum two-year followup were studied. During the study period, 193 CR TKAs were performed in 174 patients with hybrid fixation with a mean followup of 4.1 years (2–10 years), and 111 CR TKAs in 87 patients received fully cemented fixation with mean followup of 3.2 years (range 2–9 years).

The hybrid group consisted of 60 men (35%) and 114 women (65%), with mean age 59.3 (range 29–86 years). The mean BMI was 33.5 kg/m^2^ (range 18.6–53.0 kg/m^2^). The preoperative diagnosis was osteoarthritis in 184 knees (95%), posttraumatic arthritis in 6 knees (3.1%), and rheumatoid arthritis in 3 knees (1.6%). The fully cemented group consisted of 17 men (20%) and 70 women (80%), with mean age 65.9 years (range 36–85 years). The mean BMI was 35.1 kg/m^2^ (range 20.3–53.1 kg/m^2^). The preoperative diagnosis was osteoarthritis in 100 knees (90%), rheumatoid arthritis in 8 knees (7%), posttraumatic arthritis in 2 knees (2%), and other causes (pseudogout) in 1 knee (0.9%) (Table 1).

Surgery was either performed under general or spinal anesthetic. Femoral nerve and popliteal fossa blocks were used routinely from 2001 to the present. A standard medial parapatellar approach was utilized for all cases. The femoral preparation utilized intramedullary referencing with a goal of four to five degrees of valgus relative to the femoral canal for the distal cut and a standard posterior referencing guide with the goal of rotational alignment parallel to the epicondylar axis. All cuts were cooled with saline to prevent thermal damage. Femoral component sizing was based on the best fit in the anterior-posterior dimension, ensuring no medial-lateral overhang. 

The decision to use cementless femoral fixation was made intraoperatively and was based on the quality of host bone and the fit of the trial femoral component. In general, if host femoral bone was of good quality, and the trial fit was stable with no visible gaps between the bone and trial, a cementless femoral component was utilized. The implanted femoral components were either Maxim (44.6% of the hybrid knees, 55.9% of the cemented knees) or Vanguard (55.4% of the hybrid knees, 44.1% of the cemented knees) (Biomet, Warsaw, IN). Because there are small differences in femoral component design [18], the femoral component type was included as a potential confounder in the shared frailty cox regression analysis comparing survival between the two groups and was not found to be a confounding variable.

The tibial preparation was performed with an extramedullary cutting guide referencing 6–10 mm off the more normal side of the tibial plateau with an alignment goal perpendicular to the mechanical axis of the tibia. The tibial implant consisted of a modular titanium baseplate and cruciate stem with a grit-blasted titanium surface with a surface roughness average (Ra) of 6.8. After pulsatile lavage, all tibial components were cemented with either Palacos (multiple vendors) or Cobalt cement (Biomet, Warsaw, IN) mixed with 750 mg of cefuroxime antibiotic. Cement was pressurized into the cut surface of the tibia using a cement gun with a short nozzle. There was no statistical difference between the groups in terms of patellar resurfacing with 95.3% of the hybrid TKAs and 98.2% of the cemented TKAs being resurfaced.

Clinical results were graded according to the Knee Society Clinical Rating System [19]. Patients were evaluated prior to the index surgery and scheduled postoperatively at six weeks, six months, one year, and biannually thereafter. In all cases, a member of the research team other than the operating surgeon performed the clinical evaluation described above. For this study, the preoperative and most recent follow-up knee scores were compared for the clinical evaluation, as well as documentation of the number of failures, reoperations, and complications.

A weight-bearing long cassette (including hip, knee, and ankle bilaterally) lower extremity joint survey was obtained at six weeks, and anteroposterior, lateral, and sunrise radiographs were obtained at six weeks, six months, one year and biannually thereafter. For the purpose of this study, the preoperative and most recently obtained postoperative radiographs were evaluated by an experienced member of the research team other than the operating surgeon. Each radiograph was evaluated for radiolucent lines according to the Knee Society Roentgenographic Evaluation System, femoral and tibial component sagittal and coronal alignment, and overall mechanical alignment [20].

Data was analyzed by an independent statistician using commercially available software (STATA version 11-College Station, Texas, USA). The Student's *t*-test was used for comparing all continuous variables. The chi-square test was used to compare all binary variables if the expected frequencies were greater than 5. Fisher's exact test was used to compare binary variables where the expected frequencies were not adequate for the chi-square test. To account for the fact that a given patient could have more than one total knee arthroplasty in the study, which introduces a lack of independence of the observations, mixed effect regression models were used to compare knee society scores and preoperative diagnoses. Kaplan-Meier survivorship analysis was performed with failures defined as (a) revision for all aseptic causes (including component loosening, instability, extensor mechanism complications, etc.) and (b) revision for all causes. For the survival analysis, we limited our followup to 7 years, as our sample size was too small beyond this point to yield reliable survival probabilities. Shared frailty cox regression models controlling for potential confounders were used to compare survivorship between the groups. Potential confounders were identified as age, sex, diagnosis of rheumatoid arthritis, and femoral component type (Vanguard versus Maxim) as these were either significantly different between the hybrid and cemented groups (*P* < 0.05) or were nonsignificant but had a *P* value less than 0.25. A post hoc power analysis was performed given our sample sizes ARE 193 and 111. This analysis showed that we had 80% power to detect a threefold difference in failure incidence. In order to detect a smaller, more clinically relevant increase such as a 1.5-fold, the sample size would need to be too large to practically study.

## 3. Results

The preoperative knee society clinical, functional, and total scores were similar between the two groups. The mean postoperative knee society clinical score at the latest followup was higher in the hybrid group at 97 compared with a mean of 94 in the cemented group (*P* = 0.007). There was no statistically significant difference between the two groups with respect to the mean postoperative knee society functional score. The mean total postoperative knee society Score of 192 was higher in the hybrid group compared with a mean total postoperative score of 185 in the cemented group (*P* = 0.02). There was no significant difference between the two groups when comparing the change from preoperative to postoperative knee society total score (Table 2).

Radiographic followup was available in 191 knees (99%) in the hybrid group and 110 knees (99.1%) of the fully cemented controls. The mean femoral component valgus angle in the hybrid group was 5.2° (range 3.3° varus–10.4° valgus) compared with 5.8° (range 0.7° valgus–10.4° valgus) in the cemented group (*P* = 0.01). The mean femoral component flexion of 4.5° (range 0.8° extension–11.7° flexion) in the hybrid group was similar to the 4.2 of flexion (range 9.6 extension–12.4° flexion) in the cemented group. The mean tibial component varus angle of 2.0° (range 2.5° valgus–7.2° varus) in the hybrid group was similar to the 2.2 of varus (range 2.3° valgus–7.2° varus) seen in the cemented group. The two groups also had similar posterior slope, with 2.5° (range 2.6° anterior–8.8° posterior) in the hybrid group compared to 2.7° (range 1.7° anterior–10.1° posterior) in the cemented group. 

The overall number of radiolucencies seen on the femoral side was similar between the two groups with 19 (9.8%) in the hybrid group and 14 (12.6%) in the fully cemented group. The majority (12 of the 19) of the femoral-sided radiolucencies in the hybrid group was seen in Zone 1. In the cemented group, the majority of radiolucencies was seen in Zone 4 (10 of the 14), and there were significantly more Zone 4 radiolucencies in the cemented group compared to the hybrid group (10 versus 3, *P* = 0.005). No differences were seen in the number or location of tibial-sided radiolucencies between the two groups, with 6 (3.1%) in the hybrid group and 5 (4.5%) in the fully cemented group (Tables 3 and 4).

Revisions for all causes were performed in 10 of the total 193 hybrid TKAs (5.2%) and 5 of the total 111 cemented TKAs (4.5%). Revision of either the tibial or femoral component for sepsis was performed in 3 of the hybrid TKAs (1.6%) and 2 of the cemented TKAs (1.8%). Revisions of either the tibial or femoral component for aseptic causes including component loosening, instability, malalignment, or extensor mechanism-related complications were performed in 7 of the hybrid TKAs (3.6%) and 3 of the cemented TKAs (2.7%). Of all the aseptic failures, only one knee was revised for isolated aseptic loosening of the femoral component in the hybrid group. The patient, a 44-year-old female at the time of her index procedure in 1997, was revised to a cemented primary femoral component with polyethylene exchange in 2000.

**Figure 1:**
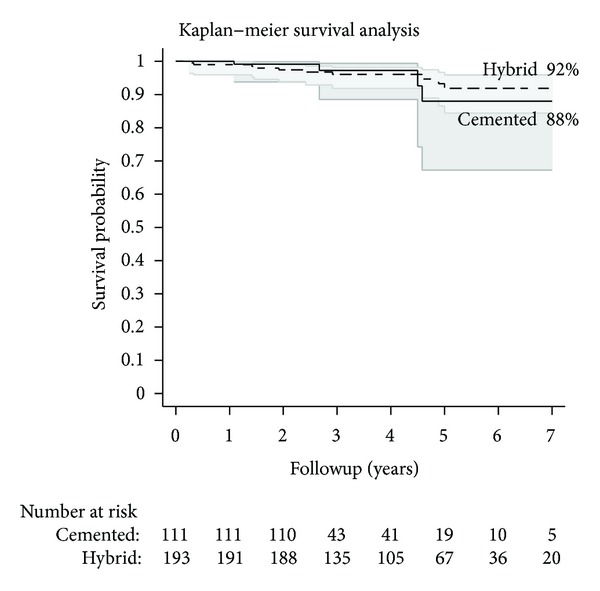
Kaplan-Meir survival curves, along with the corresponding number of patients available (“Number at risk”) for followup at the corresponding intervals, for all-cause failures are shown.

**Figure 2:**
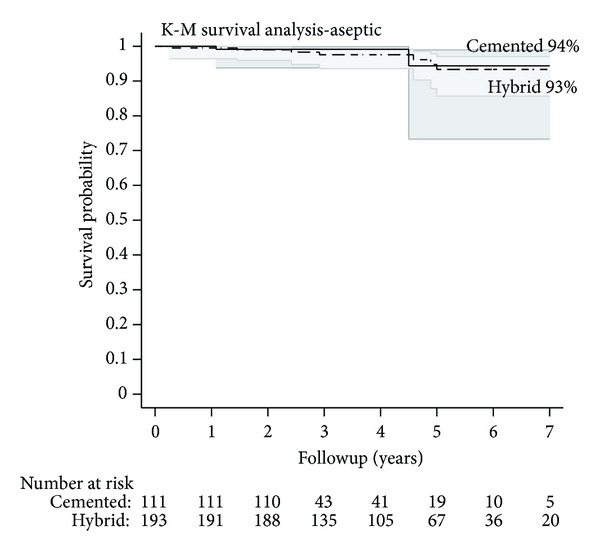
Kaplan-Meir survival curves, along with the corresponding number of patients available (“Number at risk”) for followup at the corresponding intervals, for aseptic failures are shown.

Kaplan-Meir survival curves and the corresponding number of patients available for followup at the corresponding intervals for all-cause failures and aseptic failures are shown in Figures 1 and 2. In the hybrid group, the survival rate at 2, 5, and 7 years was 97.4%, 93.3%, and 91.9%, respectively. In the cemented group, the survival rate at 2, 5, and 7 years was 99.1%, 88.0%, and 88.0%, respectively. The shared frailty cox regression models controlling for the potential confounders of age, sex, diagnosis of rheumatoid arthritis, and femoral component type did not show a statistically significant difference in survivorship between the groups in terms of all-cause failures (*P* = 0.81) or aseptic failures (*P* = 0.56).

## 4. Discussion

Potential advantages to the use of hybrid fixation in total knee replacement have been well described in previous studies, with acceptable outcomes demonstrated at short to intermediate term followup [1, 7–13, 15]. Campbell et al. previously reported on a series of 65 TKAs with 7.4-year followup demonstrating high rates of early femoral component loosening and failures [16]. In a follow-up publication, they reported on the same group of patients with an average of 15 years with continued high rates of femoral failures (11 femoral component failures out of a total of 18 failures in 65 patients) and overall survivorship of 64% at 15 years [17]. As a result of these findings, the authors had “abandoned” hybrid fixation at their institution. In response to this follow-up report, Faris et al. subsequently published their results with revision of the tibial or femoral components as the endpoint and showed 99.26% survivorship at average 7-year followup and 97.32% survivorship out to 13 years.

Based on conflicting results in the existing literature, our goal was to evaluate our own experience with hybrid fixation in total knee arthroplasty. We hypothesized that hybrid total knee arthroplasty would yield similar clinical, radiographic, and survivorship results as compared to fully cemented total knees performed over the same time period. During our study period, it was the primary surgeon's preferred method to perform hybrid fixation in those patients with adequate bone quality and coaptation of the implants during trialing. This does lead to some inherent selection bias as patients with better bone quality and likely with better overall health are selected for the study group. These selection criteria may also explain the significant differences in demographics between the study and control groups, with younger patients and more men in the hybrid fixation group. Despite this limitation, our findings demonstrate no difference in all-cause revisions between the two groups (5.2% hybrid versus 4.5% cemented, *P* = 0.79), with only one failure due to aseptic loosening of the femoral component in the hybrid group (0.5%). We did note a statistically significant difference in clinical outcome scores with higher KSS in the hybrid group, and this may also be a result of the aforementioned selection bias between the two groups. However, the overall magnitude of change from preoperative to postoperative KSS was not different between the two groups.

Additional limitations include the retrospective nature of the study and inability, due to loss of followup, to make meaningful statements about our survivorship beyond 7 years. Attempts were made to contact patients to encourage followup, but we are somewhat limited by a large referral and geographic coverage area that makes routine followup difficult. All failures in both groups of patients are accounted for to the best of our ability.

In addition to our findings being consistent with the majority of literature on hybrid fixation, we also found similar patterns in femoral-sided radiolucencies to a previous study [17]. Although our total number of femoral radiolucencies was similar between the two groups (19 (9.8%) in the hybrid group versus 14 (12.6%) in the cemented group, *P* = 0.46), and none were deemed to be radiographically loose, we did find a higher number of femoral Zone 4 radiolucencies in the cemented group (3 (1.6%) hybrid versus 10 (9%) cemented, *P* = 0.005). We believe that the higher rate of Zone 4 radiolucencies can be explained by the inability to place and pressurize cement on the posterior condyles of the femur without pushing it to the back of the knee during implant impaction and the subsequent difficulty to obtain fixation with an inadequate cement mantle at this location. The advantage of hybrid fixation with this particular issue is the allowance for bone ingrowth to occur at this location where proper cementation is difficult. With this theory, however, we are left with a persistent question as to the cause of higher femoral-sided failures and even posterior condyle fractures of the femoral components in the Mayo group's study [16, 17]. Potential factors to consider may include differences in technique or materials. The ingrowth material used in the present study as well as in the recent report by Faris et al. [14] was plasma porous spray in comparison to a beaded ingrowth surface. Osteolysis is also known to be induced by polyethylene wear. The polyethylene sterilization technique of gamma irradiation in air, which may have been used in some of the implants during the cited study, may lead to higher polyethylene wear and failure [21]. These factors may have been minimized by the use of direct compression molded polyethylene gamma irradiated in an inert environment that was used in the present study. 

We conclude that hybrid fixation leads to similar intermediate-term outcomes as fully cemented total knee arthroplasty in patients with adequate bone quality and fit of trial components and remains a viable option in total knee arthroplasty. Success may depend on implant design, ingrowth surface, patient selection, and surgical technique. Hybrid fixation may reduce the incidence of retained cement, third body wear, Zone 4 radiolucencies, and potentially late cemented component failure. Long-term followup of implants with modern materials and surgical techniques is needed.

## Figures and Tables

**Table 1:** Demographic data.

	Hybrid	Cemented	*P*value
Total no. of patients	174	87	
Mean followup (years)	4.1	3.2	****
Sex (% male)	35%	20%	0.013*
Mean age (years)	59.3	65.9	**<**0.005*
Mean height (inches)	66.9	65.1	0.08
Mean weight (lbs)	214.7	210	0.7
Mean BMI	33.5	35.1	0.41
OA	95%	90%	0.25
RA	2%	7%	0.11
PTA	4%	2%	0.46
Other diagnoses	0%	1%	0.37

Demographic data for the hybrid total knee arthroplasty and fully cemented total knee arthroplasty groups. BMI: body mass index (kg/m^2^). OA: osteoarthritis. RA: rheumatoid arthritis. PTA: posttraumatic arthritis. Other: one case of pseudogout. *Statistically significant difference (*P* < 0.05).

**Table 2:** Knee society scores (KSS).

	Hybrid	Cemented	*P* value
Total no. of knees	193	111	
Mean postop. KSS clinical	96.6	93.8	0.007*
Mean postop. KSS functional	96.6	94.9	0.22
Mean postop. KSS TOTAL	192	185.1	0.02*
Mean KSS total improvement	54.8	58.8	0.35

Clinical outcomes were measured with knee society scores (KSS) and are presented for the clinical and functional components, as well as the total score. *Statistically significant difference (*P* < 0.05).

**Table 3:** Femoral radiolucencies.

	Hybrid	Cemented	*P* value
No. of femoral radiolucencies	19 (9.8%)	14 (12.6%)	0.46
Mean radiolucency size (cm)	1.39	1.15	0.23
Femur Zone 1 lucency	12 (6.2%)	4 (3.60%)	0.33
Femur Zone 2 lucency	8 (4.2%)	1 (0.90%)	0.16
Femur Zone 3 lucency	1 (0.5%)	0	1
Femur Zone 4 lucency	3 (1.6%)	10 (9.01%)	0.005*
Femur Zone 5 lucency	0	0	
Femur Zone 6 lucency	0	0	
Femur Zone 7 lucency	0	0	

Follow-up radiographs were evaluated for radiolucencies under the femoral component. Mean size (cm) as well as the location, based on the Knee Society total knee arthroplasty roentgenographic evaluation and scoring system [19], is shown. *Statistically significant difference (*P* < 0.05).

**Table 4:** Tibial radiolucencies.

No. of tibial radiolucencies	Hybrid	Cemented	*P* value
Mean tibia radiolucency size (cm)	6 (3.1%)	5 (4.5%)	0.54
Tibia Zone 1 lucency	0.61	0.72	0.64
Tibia Zone 2 lucency	6 (3.1%)	3 (2.7%)	1
Tibia Zone 3 lucency	1 (0.5%)	0	1
Tibia Zone 4 lucency	0	0	
Tibia Zone 5 lucency	0	2 (1.8%)	0.13
Tibia Zone 6 lucency	0	0	
Tibia Zone 7 lucency	0	0	
	0	0	

Follow-up radiographs were evaluated for radiolucencies under the tibial component. Mean size (cm) as well as the location, based on the Knee Society total knee arthroplasty roentgenographic evaluation and scoring system [19], is shown. *Statistically significant difference (*P* < 0.05).
